# Epidemiological investigation and clinical presentation of severe gestational psittacosis diagnosed using targeted next-generation sequencing: A case report

**DOI:** 10.1097/MD.0000000000046860

**Published:** 2025-12-26

**Authors:** Zhichang Liu, Lixian Luo, Zewu Zhang, Xiaofang Li, Bingu Zuo, Deyuan Wu

**Affiliations:** a Dongguan Municipal Center for Disease Control and Prevention, Dongguan, Guangdong, China; b Chaonan Center for Disease Control and Prevention, Shantou, Guangdong, China; c Guangdong Field Epidemiology Training Program, Guangdong Provincial Center for Disease Control and Prevention, Guangzhou, Guangdong, China; d Dongguan Maternal and Child Health Care Hospital, Dongguan, Guangdong, China.

**Keywords:** case report, epidemiological investigation, pregnancy, psittacosis, targeted next-generation sequencing

## Abstract

**Rationale:**

Gestational psittacosis is a rare but potentially fatal cause of severe pneumonia during pregnancy. This case demonstrates how a combined epidemiological investigation and targeted next-generation sequencing (tNGS) can rapidly exclude irrelevant respiratory pathogens and enable pathogen-specific treatment.

**Patient concerns:**

A 30-year-old pregnant woman (25 weeks of gestation) presented with mild nonspecific symptoms on February 19. On February 21, she was examined at an outpatient clinic. On February 24, she was hospitalized after sudden onset of dyspnea. On admission, she exhibited severe hypoxemia and experienced rapid progression to respiratory failure with marked drowsiness.

**Diagnoses:**

Laboratory tests revealed severe anemia (hemoglobin, 78 g/L), hypoalbuminemia (14 g/L), and markedly elevated high-sensitivity C-reactive protein (144 mg/L). Chest computed tomography demonstrated multi-lobar and multi-segment consolidations in the right upper lobe and bilateral lower lobes. On day 6 after admission, tNGS of a bronchoalveolar lavage sample obtained on day 2 after clinical stabilization (day 3 after admission) identified *Chlamydia psittaci* and excluded other bacterial, viral, and fungal pathogens. Although the patient denied exposure to commercial bird markets, she had repeatedly visited a private live poultry stall.

**Interventions:**

Mechanical ventilation, anti-infective therapy comprising azithromycin and piperacillin-tazobactam, and supportive care were initiated.

**Outcomes:**

Her clinical status improved and she was discharged 14 days after admission. The patient delivered a healthy infant 71 days after discharge.

**Lessons:**

tNGS is a rapid and cost-effective tool that can exclude other pathogens in patients with gestational psittacosis. When combined with detailed epidemiological data, tNGS can guide timely pathogen-directed therapy with good maternal and fetal outcomes. Clinicians should consider exposure to private poultry sources when pregnant patients present with atypical pneumonia.

## 1. Introduction

Psittacosis is a zoonotic disease caused by *Chlamydia psittaci*, which is a pathogen that is primarily transmitted from birds, poultry, and livestock to humans.^[1,2]^ Although human-to-human spread is uncommon, some studies have indicated the potential risk of human-to-human transmission of *C. psittaci*, especially in healthcare settings and households.^[3–5]^ Clinical manifestations of human psittacosis vary from asymptomatic infections to severe complications, including severe pneumonia and death.^[6]^ During pregnancy, infection may cause serious maternal and fetal sequelae, including stillbirth, miscarriage, congenital malformations, and maternal death.^[7–9]^

Community-acquired pneumonia attributable to *C. psittaci* accounts for ~1% to 2% of all community-acquired pneumonia cases.^[10]^ However, because the clinical manifestations of psittacosis vary from asymptomatic infections to multiorgan failure,^[11]^ the treatment of numerous cases is delayed because of the lack of an early diagnosis.^[12,13]^ The reported mortality rate of severe psittacosis ranges from 10% to 20%.^[14]^ Consequently, a rapid pathogen-specific diagnostic approach is critically necessary. Metagenomic next-generation sequencing (mNGS) provides high-sensitivity microbial detection, but it is limited by its substantial cost, analytical complexity, and the need for specialized expertise.^[15,16]^ Targeted next-generation sequencing (tNGS) mitigates these limitations by focusing on a predefined panel of pathogens, thereby achieving comparable diagnostic accuracy, greater cost-effectiveness, streamlined data analyses, and shorter turnaround times.^[17]^

We present a case of gestational psittacosis complicated by severe pneumonia that was diagnosed in a timely manner using tNGS. This report illustrates the clinical utility of tNGS for rapid etiologic identification in pregnant patients with atypical pneumonia and underscores the importance of integrating epidemiological investigations, clinical assessments, and advanced molecular diagnostics to improve maternal and fetal outcomes.

## 2. Case report

On February 21, 2024, a 30-year-old pregnant Chinese woman at 25 weeks of gestation presented to HengLi Hospital, which is a public secondary general hospital in Dongguan City, Guangdong Province, with a 3-day history of fever (39°C), cough, and pain in the right lower extremity. She was initially treated with oseltamivir and cefuroxime as anti-infective therapy. However, her condition did not improve. After 2 days, the treatment regimen was modified to include piperacillin-tazobactam and azithromycin. The next day, her condition deteriorated and dyspnea worsened. Therefore, she was admitted to the emergency department of Dongguan Maternal and Child Health Care Hospital. Because her oxygen saturation (SpO_2_) was 75%, she was transferred to the medical intensive care unit for further treatment. Her medical history was unremarkable, and she had no known underlying medical conditions. She reported no recent travel history, tick bites, or acute upper respiratory tract infections. Additionally, she reported that she had not visited live bird markets and did not have friends or relatives with pet birds. Notably, she occasionally visited a private live poultry stall near her residence that was operated by her parents-in-law; however, she reported that she did not participate in any work-related activities there.

**Figure 1.**
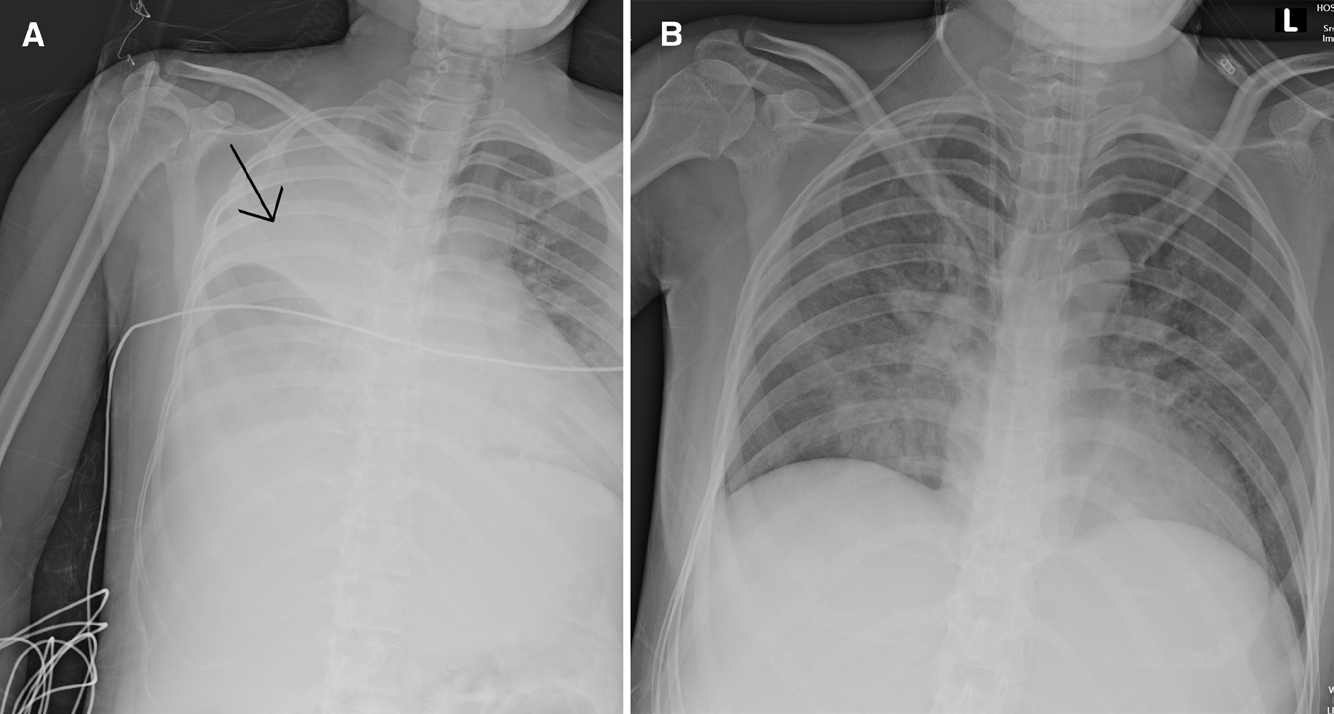
Serial chest radiographs showing interval improvement of severe pneumonia. (A) (February 25) Extensive right lung consolidation with “white-out” appearance. (B) (March 3) Marked interval resolution of bilateral pulmonary opacities.

**Figure 2.**
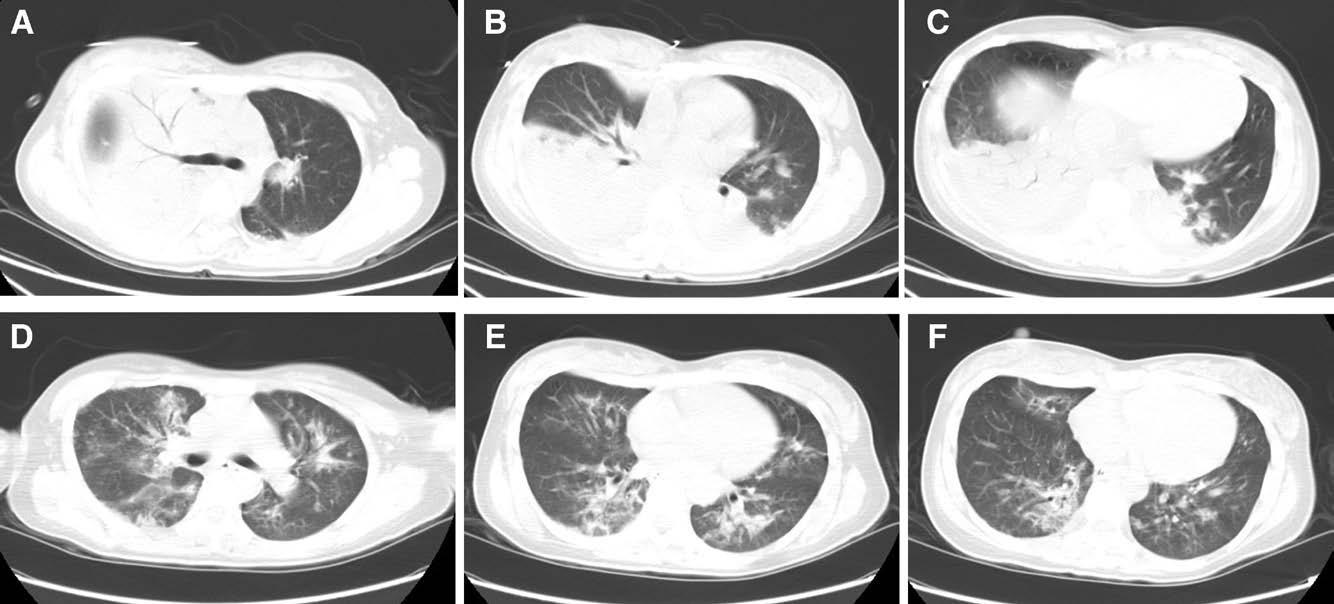
Serial chest CT scans demonstrating interval changes. (A–C) Admission CT (February 25) reveals multi-lobar consolidations in the right upper lobe and bilateral lower lobes. (D–F) Follow-up CT (March 3) shows significant resolution of the large consolidation in the right upper lobe. CT = computed tomography.

The patient presented to the medical intensive care unit with somnolence and slight cyanosis (lips and nail beds). Her vital signs were as follows: body temperature, 37.3°C; respiratory rate, 34 breaths/min; pulse rate, 124 beats/min; and blood pressure, 99/46 mm Hg. Initial blood tests revealed reduced hemoglobin (78 g/L), hematocrit (0.23) and albumin (14.00 g/L) and elevated high-sensitivity C-reactive protein (144.39 mg/L), B-type natriuretic peptide (204.80 pg/mL), and procalcitonin (2.29 ng/mL). The aspartate aminotransferase level was 72.40 U/L, urea level was 1.84 mmol/L, and uric acid level was 132.00 μmol/L. An arterial blood gas analysis revealed the following: pH, 7.47; partial pressure of oxygen, 86 mm Hg; partial pressure of carbon dioxide, 31 mm Hg; and oxygenation index, 123 mm Hg (Table 1). Routine obstetric and abdominal examinations were performed. The abdomen was soft and nontender, and the fundal height was consistent with that observed at 25 weeks of gestation. No uterine contractions were palpated, and no evidence of abdominal distension or ascites was observed. Auscultation revealed regular fetal heart tones and a heart rate of 162 beats/min. No adnexal masses or pelvic tenderness was detected. B-mode ultrasound revealed bilateral renal pelvis diameters of ~1.6 cm (right) and 1.0 cm (left), which represented mild to moderate and mild symptomatic bilateral renal pelvis dilation, respectively, with predominance on the right side. Chest radiography (Fig. 1) revealed right lung consolidation with a uniform exudative pattern. Subsequent chest computed tomography (Fig. 2) demonstrated multi-lobar and multi-segment consolidations in the right upper lobe and bilateral lower lobes. Color Doppler imaging revealed fluid accumulation in both kidneys, which was consistent with bilateral hydronephrosis. Nasopharyngeal swab SARS-CoV-2 test results were negative. Both tNGS of bronchoalveolar lavage fluid (BALF) and real-time polymerase chain reaction (PCR) confirmed the presence of *C. psittaci* 7 days after hospitalization.

**Table 1:** Biochemical analyses during the hospitalization period.

Type		Reference interval	Day 1	Day 2	Day 3	Day 4	Day 7	Day 14
WBC	10^9^/L	4.0~10.0	11.5	8.9	7.9	9.3	9.9	7.2
hs-CRP	mg/L	0~3.0	172.9	144.4	118.3	74.0	48.1	6.9
Hb	g/L	110~150	86.0	78.0	86.0	95.0	111.0	120.0
Ht	%	37~48	24.5	22.8	24.9	28.9	35.2	34.5
Plt	10^9^/L	100~300	215.0	181.0	175.0	190.0	257.0	259.0
NEUT%	%	50~75	94.8	94.0	92.3	84.9	78.8	76.8
RBC	10^12^/L	3.5~5.0	2.8	2.56	2.83	3.21	3.84	3.87
OI	mm Hg	400~500	96.0	48.0	148.0	136.0	204.0	566.0
pH	-	7.35~7.45	7.52	7.43	7.50	7.45	7.52	7.48
PO_2_	mm Hg	80~100	48.0	48.0	97.0	102.0	92.0	164.0
PCO_2_	mm Hg	35~45	29.0	33.0	27.0	37.0	45.0	33.0
BNP	pg/mL	28.3~37.5	204.8	174.9	-	-	-	-
PCT	ng/mL	0~0.5	2.3	2.1	-	-	-	-
ALB	g/L	35~55	14.0	15.0	-	23.5	34.7	31.8

ALB = albumin, BNP = B-type natriuretic peptide, Hb = hemoglobin, hs-CRP = hypersensitive C-reactive protein, Ht = hematocrit, NEUT% = neutrophil percentage, OI = oxygenation index, PCO_2_ = partial pressure of carbon dioxide, PCT = procalcitonin, Plt = platelet, PO_2_ = partial pressure of oxygen, RBC = red blood cell, WBC = white blood cell.

The patient received an anti-infective regimen comprising piperacillin-tazobactam plus azithromycin at HengLi Hospital on February 23, 2024. The fever resolved, indicating effective antimicrobial activity. However, the patient’s respiratory status remained at the peak phase of illness; therefore, the same combination was continued. A sudden decline in SpO_2_ concurrent with tachypnea occurred 23 hours later, and the patient had difficulty complying with noninvasive ventilation. The arterial blood gas test revealed the following: pH, 7.43; oxygenation index, 48 mm Hg; partial pressure of oxygen, 48 mm Hg; and SpO_2_, ~90%. After obtaining the family’s consent, the patient was immediately intubated and placed on mechanical ventilation. After emergency treatment, SpO_2_ improved and gradually reached 97%.

**Figure 3.**
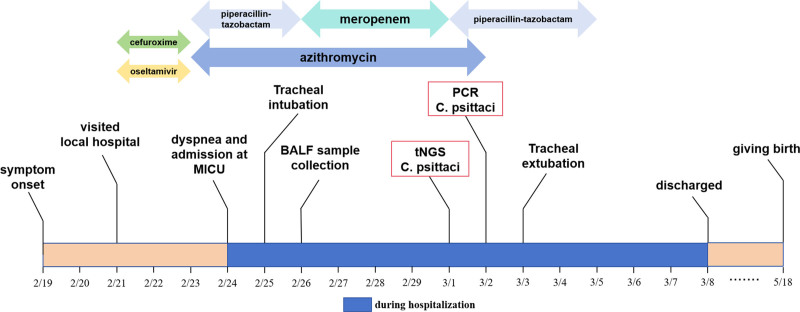
Symptom onset, and antimicrobial treatment of the gestational psittacosis case.

Ventilator-assisted breathing with tracheal intubation (days 2–9) and anti-infective therapy (azithromycin on days 1–8; piperacillin-tazobactam on days 1–3 and 7–11; meropenem on days 3–7) as well as supportive treatment including blood transfusions and albumin and globulin supplementation were administered. After tNGS of BALF suggested *C. psittaci* infection, meropenem was discontinued (Fig. 3).

After her condition improved and chest radiography showed no abnormalities, the patient was discharged on day 14 after admission (Table 1). On May 18, 2024, the patient vaginally delivered a live infant with a weight of 4.0 kg at 38 weeks of gestation. No residual organ dysfunction was observed. A physical examination of the infant revealed no congenital anomalies. Subsequently, the infant was immediately transferred to the neonatal unit for routine monitoring. The infant remained healthy, exhibited normal feeding and weight gain, and did not require respiratory support. Therefore, the infant was discharged on day 4 of life.

We used the patient’s medical records and other relevant documents to collect clinical data, including auxiliary examination results and laboratory test results, recorded during hospitalization. BALF was analyzed using tNGS at an independent reference laboratory (utilizing the Illumina NextSeq 550 platform) and real-time PCR at the Centers for Disease Control and Prevention laboratory. Environmental swabs and throat swabs obtained from household contacts were also tested using real-time PCR at the Centers for Disease Control and Prevention laboratory to determine the presence of *C. psittaci*. We performed a detailed epidemiological investigation of the patient and her family members that included individual information, clinical progression, clinical presentations, laboratory test results, history of exposure to birds, history of contact with symptomatic individuals, and history of contact with other individuals. This study was reviewed and approved by the Ethics Committee of the Dongguan Municipal Center for Disease Control and Prevention. Informed consent was obtained from the patient.

**Figure 4.**
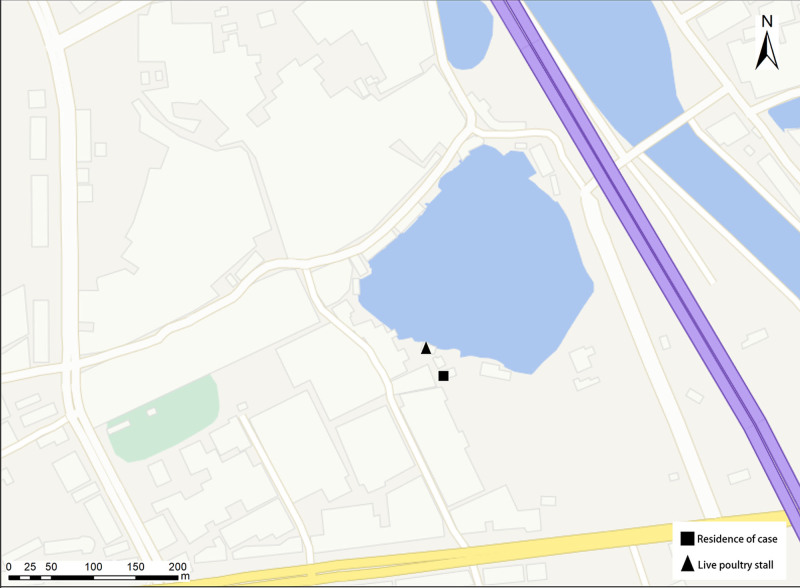
Spatial relationship between the patient’s residence and the private live poultry stall. △ = private live poultry stall; □ = patient’s residence; distance ≈ 10 m.

The patient resided with 5 family members (husband, daughter, father-in-law, and mother-in-law) in a one-story house in the countryside. The patient’s parents-in-law managed a private live poultry stall where processing and slaughtering of live poultry was performed for local residents. The stall was situated within a 10-m radius of the patient’s residence and had an area of 120 m^2^ (Fig. 4). Inside the stall, 3 coops housed 35 chickens and 10 geese. Although the patient did not work at the stall, she occasionally walked within its vicinity. Environmental sampling included swabs of the exterior of the patient’s home and the poultry stall (including poultry waste, equipment, and capes), and throat swabs were obtained from 4 family members. However, none of the samples exhibited positive real-time PCR results for *C. psittaci*.

## 3. Discussion

### 3.1. Summary of the case

Our case report describes a pregnant woman presented with a progressive respiratory illness that culminated in severe hypoxemia and respiratory failure. tNGS of a BALF sample identified *C. psittaci*, thereby excluding other bacterial, viral, and fungal pathogens. She received a combination of piperacillin-tazobactam and azithromycin, was placed on mechanical ventilation, and received comprehensive supportive care, leading to a favorable maternal and fetal outcome.

To ascertain the novelty of our report, we performed a systematic PubMed search of (“psittacosis” OR “chlamydiosis” OR “C. psittaci”) AND (“pregnancy” OR “pregnant” OR “gestation” OR “antenatal”) AND (“targeted next-generation sequencing” OR “tNGS”) from inception until August 2025 yielded no previously published cases, thus highlighting the novelty of our findings.

### 3.2. Differential diagnosis and the role of molecular testing

Potential sources of *C. psittaci* infection are almost exclusively linked to close contact with infected birds, particularly the inhalation of aerosols generated from bird urine, feces, and other excretions.^[6]^ The initial presentation (cough, fever, and subsequent dyspnea) of our patient indicated classic psittacosis, but it also closely resembled that of many common respiratory infections (cough, fever, muscle aches, and gastrointestinal symptoms)^[5,18,19]^; therefore, the misdiagnosis rate of such cases is as high as 50%.^[20]^ Because the patient’s procalcitonin level was only slightly elevated and no obvious systemic signs were observed, the likelihood of a typical bacterial infection was low. However, her history of contact with poultry raised the possibility of *C. psittaci* or avian influenza.

Clinical symptoms and chest radiography alone are insufficient for distinguishing these entities. Although lung consolidation on chest radiography is not a typical manifestation of avian influenza virus pneumonia, patients with certain progressive cases, and especially those with H7N9, may exhibit such radiological changes.^[21,22]^ Therefore, etiological detection is the most effective method of differentiation. A comparison of the clinical, radiographic, and epidemiological features of psittacosis and avian influenza is presented in Table 2.

**Table 2:** Differential diagnosis: avian influenza vs psittacosis.

Feature	Psittacosis	Avian influenza (e.g., H5N1, H7N9)
Causative agent	*Chlamydia psittaci*	Avian influenza virus
Typical exposure	Birds (parrots, pigeons, poultry, etc) with healthy or diseased appearance	Contact with sick/dead birds, live poultry markets, contaminated environments
Incubation period	5–14 d (range 3–45 d)	2–5 d (usually < 7 d)
common clinical manifestations	Fever, headache, dry cough, myalgia, fatigue	Fever, cough, sore throat, myalgia, conjunctivitis
Radiological findings (chest CT)	Unilateral or multifocal, patchy or lobar consolidation	Bilateral, diffuse (often from central lobe segments outward)
Pregnancy safety	Azithromycin preferred; doxycycline contraindicated	Oseltamivir is category B

CT = computed tomography.

Since the development of mNGS, psittacosis has become increasingly recognized and reported in recent years.^[12,23]^ Despite the promising applications of mNGS in microbiological and clinical diagnostics, its widespread adoption has been impeded by several challenges, including its high cost, difficulty distinguishing colonization from true infection, and the need for highly skilled personnel.^[24]^ Compared with mNGS, tNGS has several advantages, including higher sensitivity, cost-effectiveness, and relatively simple data analyses.^[25]^ The results of tNGS and mNGS are generally comparable when diagnosing severe pneumonia.^[26]^ Although tNGS has limitations when used to identify new pathogens, its effectiveness is significantly enhanced when combined with epidemiological investigations such as occupational histories and animal contact histories, thereby improving the diagnostic capabilities for psittacosis cases. A comparison of mNGS, tNGS, and PCR is provided in Table 3.

**Table 3:** Comparative performance of real-time PCR, tNGS, and mNGS.

Feature	tNGS	mNGS	PCR
Principle	mp-tNGS: Multiple PCR + NGS hc-tNGS: probe capture + NGS	Undifferentiated high-throughput sequencing	Targeted amplification
Turnaround time	Moderate (10–24 h)	Long (17–48 h)	Short (1–4 h)
Cost per sample	Moderate (mp-tNGS: $100–$200, hc-tNGS: $200–$500)	High ($500–$1000)	Low ($50–$100)
Sensitivity for conventional microbiological	High	Moderate	Very high
Personnel expertise required	High (standard NGS workflow)	Very high (complex library prep & bioinformatics)	Low
Detection breadth	Predefined broad	Extremely wide	Single
Novel pathogen discovery	No	Yes	No
Key advantage	Breadth, sensitivity balance	Unbiased, broadest detection	Speed, sensitivity, cost
Key limitation	Limited to panel	Cost, complexity, background noise	Narrow scope

hc-tNGS = hybrid capture-based tNGS, mNGS = metagenomic next-generation sequencing, mp-tNGS = multiplex PCR-based tNGS, PCR = polymerase chain reaction, tNGS = targeted next-generation sequencing.

Although PCR is fast and inexpensive, it is heavily dependent on the expertise of the clinician because it involves a single target. Epidemiological clues are often equivocal. For example, bird exposure may indicate *C. psittaci*, but it also could indicate avian influenza, *Salmonella* spp., or other zoonotic agents; therefore, coinfections must be considered. Moreover, psittacosis is uncommon in routine practice, and many hospitals lack relevant PCR assays. In contrast, tNGS does not rely on prevalidated primers and can simultaneously screen for a broad spectrum of pathogens, thus providing a more comprehensive diagnostic approach when the etiology is uncertain. In our case, because of the underdevelopment of local tNGS services, the entire laboratory workflow from specimen collection to final report release required ~96 hours (~1 day for sample transport and ~3 days for laboratory processing and report generation). The workflow analysis identified the following 2 primary bottlenecks: the time required to transport specimens from the bedside to the reference laboratory, and downstream sequencing and bioinformatics pipelines. Further refinement of the tNGS workflow could enable clinically actionable results within 24 hours.

### 3.3. Therapeutic strategy

An epidemiological assessment and rapid molecular diagnosis are essential to precision antimicrobial stewardship during pregnancy. These factors allow clinicians to swiftly select the most appropriate pregnancy-safe drug based on the exposure history and simultaneously rule out erroneous empirical choices and exclude infection by other potential pathogens. This case demonstrates the impact of tNGS-guided antimicrobial stewardship on severe pneumonia. The initial empirical therapy comprising piperacillin-tazobactam and azithromycin was escalated to meropenem and azithromycin after intubation for severe pneumonia. After tNGS confirmed *C. psittaci*, therapy was de-escalated. Meropenem was discontinued because it was ineffective against psittacosis, whereas piperacillin-tazobactam as bacterial coinfection prophylaxis was retained with targeted azithromycin therapy.

To determine the optimal antimicrobial for our pregnant patient, we evaluated the safety and efficacy of potential options. Although doxycycline is a well-established first-line agent for psittacosis,^[27]^ it is a tetracycline that is contraindicated during pregnancy because of its propensity to cause permanent fetal tooth discoloration and skeletal dysplasia.^[28–30]^ Azithromycin, which is a macrolide, is regarded as a category B drug with a substantially safer profile during gestation.^[7,31]^ Although some studies have raised concerns about the potential effects of azithromycin on ovarian development, evidence supporting its use for pregnant patients with *Chlamydia* infections has remained overwhelmingly positive.^[32]^ Published case reports and small case series of pregnant women with *C. trachomatis* or *Chlamydia pneumoniae* infections treated with azithromycin have described complete maternal cure and normal neonatal outcomes.^[33,34]^ Although the literature that specifically addressed macrolide therapy for psittacosis during pregnancy is limited, no adverse fetal outcomes that were directly associated with psittacosis treatment have been reported in the available case series.^[35]^

### 3.4. Prognosis

According to previous reports, *C. psittaci* infection during pregnancy can lead to severe consequences, including atypical pneumonia, sepsis, diffuse intravascular coagulation, hepatic dysfunction, and renal dysfunction.^[7]^ Our patient’s condition rapidly deteriorated, and respiratory failure, pleural effusion, bilateral hydronephrosis, and hypoproteinemia developed. However, because of the gestational age and physiological changes in the urinary tract associated with pregnancy, we believe that this case was physiological hydronephrosis during pregnancy rather than acute kidney injury caused by infection. Clinical and laboratory evaluations revealed no underlying immunological risk factors, including diabetes mellitus, malignancy, chronic kidney disease, immunosuppressive therapy, and human immunodeficiency virus infection.

The predominant driver of disease was exposure to infected birds rather than an intrinsic immune defect. Prompt initiation of a dual-agent regimen comprising azithromycin (a pregnancy-safe macrolide) and piperacillin-tazobactam (to prevent potential bacterial coinfections) resulted in favorable maternal and fetal outcomes.

### 3.5. Public health implications

Our patient did not have any previously described immunological risk factors for *C. psittaci* infection. Additionally, she did not have close contact with parrots or other exotic birds. However, according to some studies, *C. psittaci* infection is associated with occupational exposure to poultry.^[36]^ Notably, transmission can occur not only between poultry with infections and their close contacts but also between those with secondary and tertiary infections and their close contacts. Zhang^[3]^ reported that some close contacts of asymptomatic carriers exhibited respiratory symptoms and subsequently received positive test results for *C psittaci*. These findings underscore the need to give greater attention to potential human-to-human transmission of psittacosis among medical staff and other close contacts, particularly pregnant women. Although we did not detect any positive test results among the samples from our patient’s family members or the external environment of the house and poultry stall using real-time PCR, the patient’s recent activity history led us to believe that poultry were the most probable source of infection. However, confirming the specific route of infection remains challenging. Infection could have occurred through poultry, human-to-human transmission from her parents-in-law who worked with poultry, or other undiscovered pathways. Although no positive cases have been detected in chickens, some studies reported that positive samples can be found in the external environment and among farm workers.^[37]^ Pregnant women should avoid contact with live poultry and places where poultry are sold.

When patients present with unexplained flu-like illnesses, chest radiography, testing to detect hepatic dysfunction, renal dysfunction, or elevated CRP and procalcitonin levels,^[38,39]^ and obtaining an exposure history (including contact with parrots, goats, or poultry) are essential. Early macrolide therapy can improve maternal and fetal outcomes. Prompt collection of peripheral blood or BALF is crucial. Throat swabs may miss lower respiratory tract infections.^[40]^ Therefore, both mNGS and tNGS are recommended for diagnosing psittacosis; however, the patient’s financial situation should be considered before choosing between mNGS and tNGS. Additionally, comprehensive epidemiological evidence strongly supports the validity of tNGS.

### 3.6. Limitations

One limitation of this study was that we were unable to conduct blood antibody tests of the family members of the patient and the newborn because of the lack of antibody reagent tests for *C. psittaci*. Moreover, environmental sampling was delayed until 8 days after hospitalization. Ongoing operations and cleaning activities likely disturbed contamination of the poultry stall, and warm, dry conditions likely accelerated pathogen inactivation and reduced environmental sampling sensitivity.

## 4. Conclusions

This case that was diagnosed using tNGS illustrates the pivotal role of rapid molecular testing in identifying atypical pathogens and guiding timely and safe therapy during pregnancy. A detailed epidemiological interview revealed the patient’s repeated visits to a private live poultry stall. This avian exposure was the main clue that led the obstetric team to empirically start a pregnancy-safe macrolide (azithromycin) long before the tNGS results were available. Early macrolide-based therapy prevented further respiratory decline and was a decisive factor in achieving favorable maternal and fetal outcomes. Additionally, this case illustrated that the integration of astute clinical assessments, including a thorough exposure history, with rapid molecular diagnostics can direct timely, life-saving interventions in complex obstetric scenarios, thereby potentially reducing the use of unnecessary broad-spectrum antibiotics and informing public health measures.

## Acknowledgments

The authors thank the staff of the Institute of Infectious Disease Prevention and Control, as well as the Clinical Laboratory affiliated with the Dongguan Municipal Center for Disease Control and Prevention. Additionally, the authors thank Zhang Meng for his valuable suggestions regarding the manuscript.

## Author contributions

**Data curation:** Lixian Luo.

**Funding acquisition:** Zewu Zhang.

**Investigation:** Zhichang Liu, Lixian Luo, Xiaofang Li, Bingu Zuo, Deyuan Wu.

**Methodology:** Lixian Luo, Xiaofang Li, Bingu Zuo.

**Project administration:** Zewu Zhang.

**Resources:** Xiaofang Li.

**Supervision:** Zewu Zhang, Xiaofang Li.

**Validation:** Bingu Zuo, Deyuan Wu.

**Visualization:** Zhichang Liu.

**Writing – original draft:** Zhichang Liu, Lixian Luo.

**Writing – review & editing:** Zhichang Liu, Zewu Zhang.
